# Prescription Pattern of Laxatives for Opioid-Induced Constipation in Japanese Patients With Chronic Non-cancer Pain: A Retrospective Cohort Study of a Health Insurance Claims Database

**DOI:** 10.7759/cureus.78212

**Published:** 2025-01-29

**Authors:** Motoki Sonohata, Misato Kitamura, Akira Hashimoto, Yasuhide Morioka

**Affiliations:** 1 Japan Community Healthcare Organization, Saga Central Hospital, Saga, JPN; 2 Medical Affairs Department, Shionogi & Co. Ltd., Osaka, JPN

**Keywords:** health insurance claims database, laxatives, non-cancer pain, opioid-induced constipation, weak opioids

## Abstract

Introduction

Opioid-induced constipation (OIC) is the most common side effect of opioids. Weak opioids are often prescribed for non-cancer chronic pain in Japan; however, data on laxative use are scarce. We evaluated the real-world prescription patterns of laxatives for OIC in patients administered weak opioids for non-cancer pain in Japan.

Methods

In this retrospective cohort study, we extracted data from a Japanese health insurance claims database for patients with chronic non-cancer pain (lower back pain, joint pain, and neuropathic pain) who were continuously prescribed weak opioids for ≥1 month after initiating weak opioids between 2017 and 2022.

Results

Of 425,556 patients prescribed weak opioids, 42,514 patients (mean age: 50.1 years) were included. Most were prescribed ≤150 mg tramadol equivalent/day, and 21,405 (50.3%) were on weak opioids for lower back pain. The proportion of laxatives prescribed on the first day of weak opioid prescription was 6.7%; the cumulative prescription rate during the 12‑week follow-up was 13.6%. The most common laxatives during the first week were magnesium oxide (2,320 (66.6%)), sennoside (484 (13.9%)), and naldemedine (301 (8.6%)); at 12 weeks, these proportions were 55.2%, 13.1%, and 13.8%, respectively.​ The amount of weak opioids prescribed to patients without laxatives was lower than the standard dosage and was also lower than the amount prescribed to patients with laxatives.

Conclusions

This study showed that the actual prescription patterns of laxatives after initiating weak opioids in patients with chronic non-cancer pain were variable, depending on the duration after initiating weak opioids. The association between laxative use and weak opioid dosage indicates that OIC management may have a potential impact on pain management.

## Introduction

Globally, pain is considered a significant public health problem, and 20% of adults suffer from it [1]. The prevalence of chronic pain (pain lasting ≥3 months) in Japanese adults has been reported to be 39.3% [2]. Non-cancer-related chronic pain can have behavioral, psychological, and societal impacts on individuals and can lead to loss of activity and isolation [3,4]. Musculoskeletal pain, neuropathic pain, and functional pain syndrome are among the most common types of non-cancer-related chronic pain [5]. Opioids, initially developed for cancer pain, are being increasingly used and are one of the most prescribed classes of drugs in non-cancer-related chronic pain management [6,7].

Although opioids are effective for non-cancer pain management, they can cause adverse effects and treatment discontinuation [8-10]. Patients experience severe adverse events related to the gastrointestinal system [10], of which opioid-induced constipation (OIC) is the most common [10,11]. OIC is characterized by difficulty passing stools, the passage of hard stools, and a sense of incomplete bowel emptying or anorectal obstruction [12]. Opioids activate the µ-opioid receptors and reduce peristalsis and motility. They increase fluid absorption, causing incomplete stool emptying [13]. Opioids also increase the anal sphincter tone, which contributes to anal blockage and constipation [14]. In Japanese patients using opioids for non‑cancer chronic pain, the incidence of constipation has been reported as 34% [15].

Laxative use is the preferred first-line pharmacological approach to managing OIC [16]. In both the American Gastroenterology Association guidelines and the European expert consensus statement, traditional laxatives are recommended as the first-line treatment, followed by peripheral μ-opioid receptor antagonists (PAMORAs) as the second-line treatment for OIC [17,18]. In Japan, the simultaneous administration of osmotic agents or stimulant laxatives with opioids is recommended, and PAMORAs are recommended in cases where the first-line treatment is ineffective [19].

A Japanese study describing opioid use in cancer pain and laxative use in OIC management in patients with cancer reported that 63.3% of patients with cancer were prescribed laxatives following the prescription of weak opioids [20]. However, the real-world evidence on laxative prescription patterns in Japanese patients with OIC with non-cancer pain is limited. The primary objective of this study was to evaluate the real-world prescription patterns of laxatives in patients with OIC who were prescribed weak opioids for chronic non-cancer pain in Japan. The secondary objective was to assess how laxative use correlated with weak opioid dosage and to identify trends over the first 12 weeks of treatment.

## Materials and methods

Study design

This was a retrospective cohort study using a Japanese health insurance claims database provided by JMDC Inc. (JMDC), a unique database from Japan's health insurance societies, which primarily consists of patients aged <65 years [21], including ledgers of insureds, claims (for hospitalization, outpatient treatment, drug preparation, and dental treatment), and health checkup results [22]. The database comprises approximately 19 million insureds as of March 2024 [22].

Data encoded within the database between January 2017 and June 2022 were extracted. We prepared master forms for receipt data and numerical information for disease diagnosis (International Classification of Diseases, Tenth Revision (ICD-10) code (https://iris.who.int/handle/10665/42980)), prescription drugs (Anatomical Therapeutic Chemical (ATC) classification system code (https://www.who.int/tools/atc-ddd-toolkit/atc-classification)), medical practice, and drug units that had been standardized. Medical institution names were replaced with unique IDs in the database.

Study population

The index date was the date of the first prescription of weak opioids. We included male and female patients with no diagnosis of malignancy during the baseline period (i.e., three months preceding the index date, excluding the month of the index date) and follow-up period (i.e., four months after the index date, including the month of the index date); who had been newly prescribed weak opioids for lower back pain (using ICD-10 codes listed in Appendix A: M40-M43, M45-M49, M50-M54), joint pain (M05-M14, M15-M19, M20-M25, M60-M63, M70-M79), and neuropathic pain (G98, G64, G969, E144, B02, G50-G59, G60-G64) for at least one month from the index date; and for whom data were available for three months before and four months after the index date.

If the period from the end of the prescription of the weak opioid to the next prescription date (next prescription date minus prescription end date) was seven days, and a repeat prescription fell within this range, it was considered a continuous prescription. Patients who were prescribed weak opioids during the follow-up period were also included.

Patients with a diagnosis of malignant tumor (C00-C26, C30-C41, C43-C58, C60-C97, D00‑D07, D09) and muscle pain (M791) were excluded. In addition, patients were excluded if they were prescribed laxatives or strong opioids during the baseline period, or if they were prescribed weak opioids at baseline but switched to strong opioids during the follow-up period. The study excluded patients who had been taking laxatives before the use of weak opioids; hence, we defined OIC as occurring in patients who were newly prescribed laxatives after starting their prescription of weak opioids.

Outcomes

The outcomes included the prescription rate of laxatives for 12 weeks from the index date, the prescription ratio by type of laxatives for 12 weeks from the index date, and the amount of weak opioids prescribed to patients with or without laxative prescriptions after the index date.

Measurements

Baseline Variables

The data collected retrospectively included age, sex, pain classification during baseline, prescription of laxatives, and the prescription amount of weak opioids per day.

Codeine (R05D1), tramadol (N02B), tramadol/acetaminophen (N02B), and pentazocine (N02B) were categorized as weak opioids. In contrast, oxycodone (N02A), tapentadol (N02A), hydromorphone (N02A), fentanyl (N02A/N01A2), buprenorphine (N02B), methadone (N02A), and morphine (N02A) were categorized as strong opioids per the Japanese clinical guidelines for pain management [19]. The laxatives included magnesium oxide (A02A1), sennoside (A06A2), sodium picosulphate (A06A2), naldemedine (A06A9), lubiprostone (A06A9), lactulose (A06A6), polyethylene glycol (A06A7), other osmotic laxatives (A06B2), bisacodyl (A06A2), senna (V03B2/A06A2), linaclotide (A03G), kampo (V03B), elobixibat (A06A9), glycerin enema (A06A4), and other laxatives listed in ATC A06A except the laxatives mentioned above.

Statistical analyses

The study included data from all patients in the JMDC database who fulfilled the eligibility criteria. Missing data were not imputed. Patients without a prescription date were treated as having missing data and were not included in the analysis. Demographic data (age, sex, pain classification during the baseline period) were tabulated.

Descriptive statistics were used for analyses. The number and proportion of patients prescribed laxatives for 4, 8, and 12 weeks after the index date; the number and proportion of patients prescribed at least one type of laxative; and the cumulative number of patients prescribed laxatives were tabulated. In addition, the prescription rate of laxatives by the daily prescription amount of weak opioids was tabulated for 4, 8, and 12 weeks after the index date. A Kaplan-Meier curve was plotted to depict the time to the first laxative prescription during the study period.

The patients were stratified into those who had been prescribed laxatives at least once after the index date, and those who had never been prescribed a laxative. The daily rate of weak opioid prescriptions from the index date to the last day of prescription was then calculated for both groups. The median daily prescription and interquartile range (IQR) of weak opioids were presented as a box plot for patients with or without laxative use. The prescription end date was defined as the prescription start date plus the number of prescription days minus one. If the prescription for weak opioids ended after the fifth week for a patient, it was considered as study discontinuation.

The daily prescription amount of weak opioids was calculated by dividing the total amount prescribed during the number of consecutive days by the number of consecutive days of prescription. Based on the equivalent amount of oral morphine hydrochloride hydrate, the amount of tramadol was calculated as a ratio of tramadol hydrochloride: codeine phosphate hydrate: pentazocine hydrochloride = 150 mg: 200 mg: 170 mg, and all values were converted to tramadol hydrochloride. The calculation assumed that one tramadol hydrochloride/acetaminophen combination tablet contains 37.5 mg of tramadol hydrochloride [23,24].

The statistical analysis was performed using SAS version 9.4 (SAS Institute, Cary, North Carolina, US).

## Results

Population analysis

**Figure 1:**
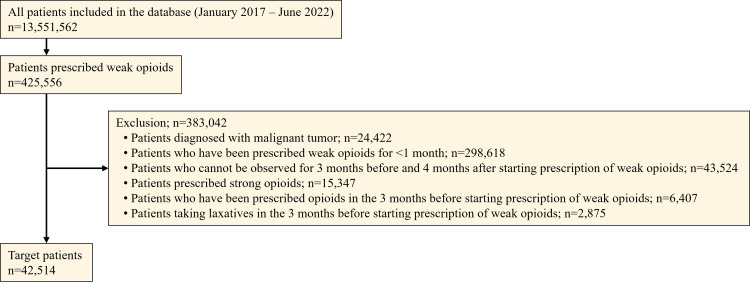
Patient flowchart n, number of patients

Of the 13,551,562 individuals recorded between January 2017 and June 2022 in the JMDC database, 425,556 were prescribed weak opioids. Of these, a total of 42,514 patients were included in the study. The population flow is shown in Figure 1.

The reasons for patient exclusion were diagnosis of malignancy (n=24,422) and prescription of weak opioids for <1 month from the index date (n=298,618). For 43,524 patients, the data could not be obtained for three months before and four months after the index date. A total of 15,347 patients were excluded for strong opioid prescriptions during the study period. In 6,407 patients, weak opioids were prescribed three months before the index date, and 2,875 patients were on laxatives three months before the index date (Figure 1).

The mean age of the cohort was 50.1 years, and 24,929 (58.6%) patients were male (Table 1). The majority of participants were in their 50s (15,469 (36.4%)), followed by the 40s (11,572 (27.2%)) and >60 years of age (8,519 (20.0%)) (Appendix B). Half of the patients (21,405 (50.3%)) were on weak opioids for lower back pain (Table 1).

**Table 1:** Patient demographics and pain categories ^*^Percentages were calculated horizontally ^†^Multiple answers were permissible n, number of patients

Patient characteristics	All patients	Patients prescribed laxatives for 12 weeks after the index date*
Total number, n (%)	42,514 (100)	5,772 (13.6)
Mean age (years)	50.1	51.2
Sex, n (%)		
Female	17,585 (41.4)	2,834 (16.1)
Male	24,929 (58.6)	2,938 (11.8)
Disease^†^, n (%)		
Lower back pain	21,405 (50.3)	2,961 (13.8)
Joint pain	16,236 (38.2)	2,085 (12.8)
Neuropathic pain	12,800 (30.1)	1,885 (14.7)

Patterns of laxative prescription

**Figure 2:**
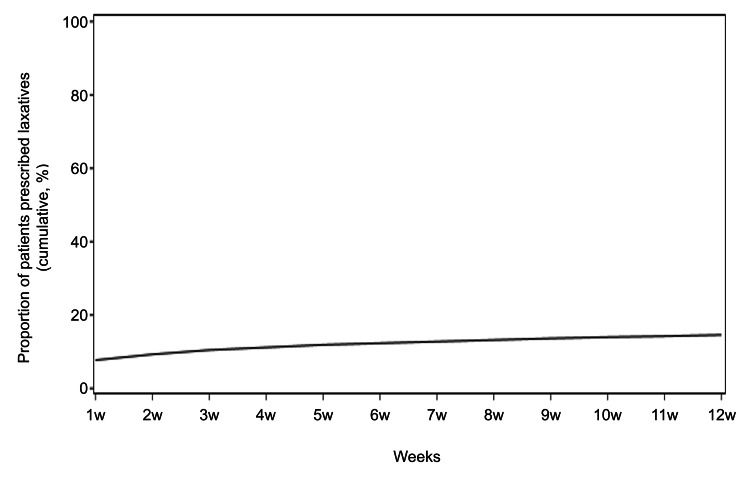
Cumulative laxative prescription rate in patients receiving weak opioids: the proportion of patients prescribed at least one type of laxative during the 12 weeks after initiating weak opioids

The mean age of patients prescribed laxatives was 51.2 years. The overall prescription of laxatives in patients administered weak opioids was 13.6% during the 12 weeks from the index date, and female patients had higher prescriptions of laxatives (16.1%) than male patients (11.8%) (Table 1). The total prescription rate of laxatives was 6.7% on day 1; the cumulative prescription rate for laxatives (i.e., the percentage of patients prescribed at least one type of laxative during the 12 weeks after initiating weak opioids) was 13.6% (Figure 2).

Overall, most of the patients were prescribed ≤150 mg tramadol equivalent/day. The proportion of laxative use tended to increase as the amount of weak opioids prescribed increased. This trend was observed as early as four weeks (from 9.3% for 0-≤50 mg/day to 31.4% for >300 mg/day).​ The proportion of laxative use increased over time, but the rate of increase was gradual​ (Table 2).

**Table 2:** Quantity of weak opioids prescribed to patients with or without laxative use by each week of follow-up ^*^Percentages were calculated horizontally n, number of patients

Tramadol equivalent, mg/day	Patients, n (%)	Laxative users, n (%)*
Total	42,514 (100.0)	-
4 weeks		
0-≤50	11,825 (27.8)	1,103 (9.3)
>50-≤100	15,247 (35.9)	1,557 (10.2)
>100-≤150	12,712 (29.9)	1,615 (12.7)
>150-≤200	1,964 (4.6)	370 (18.8)
>200-≤250	498 (1.2)	105 (21.1)
>250-≤300	198 (0.5)	42 (21.2)
>300	70 (0.2)	22 (31.4)
8 weeks		
0-≤50	4,374 (10.3)	444 (10.2)
>50-≤100	8,434 (19.8)	1,149 (13.6)
>100-≤150	7,241 (17.0)	1,167 (16.1)
>150-≤200	1,419 (3.3)	316 (22.3)
>200-≤250	411 (1.0)	112 (27.3)
>250-≤300	136 (0.3)	38 (27.9)
≥300	49 (0.1)	17 (34.7)
12 weeks		
>0-≤50	2,326 (5.5)	286 (12.3)
>50-≤100	5,325 (12.5)	804 (15.1)
>100-≤150	4,535 (10.7)	838 (18.5)
>150-≤200	968 (2.3)	257 (26.5)
>200-≤250	329 (0.8)	95 (28.9)
>250-≤300	95 (0.2)	33 (34.7)
≥300	32 (0.1)	7 (21.9)

**Figure 3:**
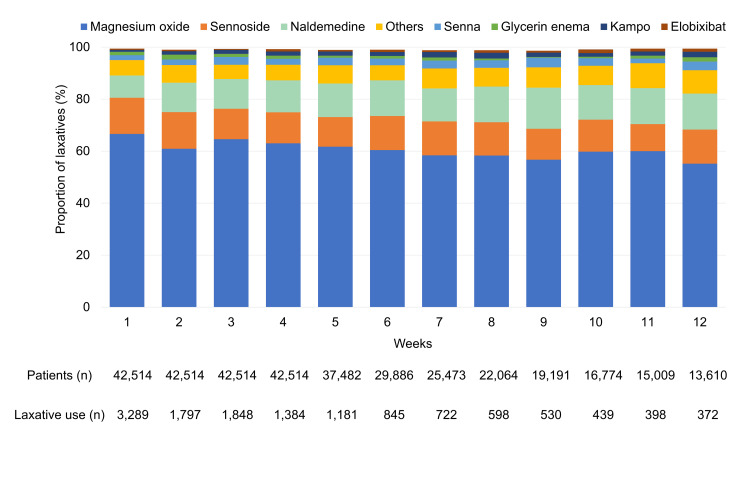
Prescription rate of laxatives by type n, number of patients Laxatives prescribed to <1% of patients: bisacodyl, lactulose, linaclotide, and stimulant laxatives Laxatives prescribed to 0% of patients: picosulphate, lubiprostone, polyethylene glycol, and other osmotic laxatives Others: other laxatives in ATC code A06A

At week 1, magnesium oxide was the most commonly prescribed laxative (2,320 (66.6%)), followed by sennoside (484 (13.9%)) and naldemedine (301 (8.6%)). The proportion of prescriptions for magnesium oxide gradually decreased to 55.2%, while that of naldemedine gradually increased to 13.8% through 12 weeks. The proportion of other laxatives increased from 5.9% in week 1 to 9.0% in week 12 (Figure 3, Appendix C). During the entire 12-week period, the laxatives bisacodyl, lactulose, linaclotide, and other stimulant laxatives were prescribed to <1% of patients while sodium picosulphate, lubiprostone, polyethylene glycol, and other osmotic laxatives were prescribed to 0.0% of patients.

**Figure 4:**
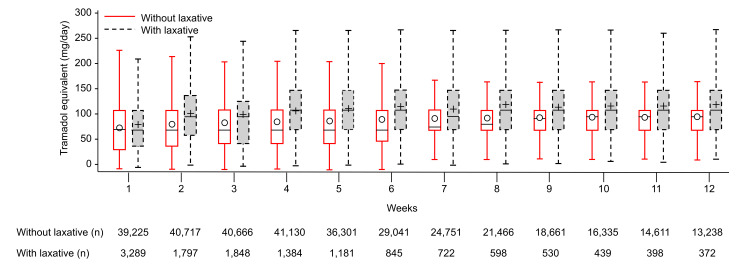
Comparison of weak opioid quantities prescribed to patients with or without laxative use mg, milligrams; n, number of patients Note: The box plot is drawn from the first- to the third quartile. The horizontal line within the box plot represents the median value while the symbols (ο and +) represent the mean value. The lower and upper whiskers represent the minimum and maximum values, respectively.

During the first week after the index date, the median (IQR) amount of weak opioids prescribed was similar in groups with (75.0 (45.0-112.5) mg/day) and without laxative use (75.0 (37.5-112.5) mg/day). However, from the second week onward, the amount of tramadol was substantially higher in patients with laxative use (100.0 (64.3-139.3) mg/day) than in those without laxative use (75.0 (45.0-112.5) mg/day) (Figure 4).

## Discussion

This was the first Japanese health insurance claims database study to ascertain laxative use in patients receiving weak opioids for non-cancer chronic pain.

We found that over 12 weeks, the cumulative laxative prescription rate in patients receiving weak opioids was 13.6%. As for the prescription rate, it was lower than the rate previously reported (33%) in a questionnaire survey in a similar Japanese cohort [15]. However, the survey included patients who had been taking opioids for >3 months, who were using laxatives before opioids were prescribed, and included 11% of patients who were prescribed strong opioids.

Laxative use among patients using weak opioids for non-cancer chronic pain seems to be different from its use in those using weak opioids for cancer pain. In Japanese patients with cancer who were receiving weak opioids, 63.3% were prescribed laxatives during the 3-month follow-up period [20]. Among recipients of weak opioids, the proportion of patients with cancer who received laxatives early (i.e., within three days of opioid prescription) was substantially higher in hospitalized patients compared with outpatients [20]. Healthcare practitioners may frequently ask hospitalized patients about their constipation, or patients may immediately volunteer the information. Notably, in the current study, the number of patients who received weak opioid prescriptions decreased over time. It is known that OIC persists with long-term opioid use, even in patients using laxatives [20]. To avoid associated side effects, including OIC, most patients are likely to discontinue the use of weak opioids. However, from another perspective, it is not desirable that weak opioids are discontinued by patients for the management of pain. Instead, by properly managing the side effects of weak opioids, including OIC, it may be possible to prevent patients from discontinuing or reducing the dosage of weak opioids.

Unsurprisingly, female patients (2,834 (16.1%)) had higher laxative prescriptions than male patients (2,938 (11.8%)) in this claims database study. As shown previously in a questionnaire-based French study, female patients were more likely to develop OIC, with the prevalence of constipation being 71.0% in female patients compared with 28.7% in male patients [25]. The higher laxative prescription rates observed in women may be related to the known sex-based differences in the prevalence of constipation and opioid-induced side effects. Specifically, women are more likely to report constipation [26], although further research is required to understand the underlying causes.

Our results demonstrate the tendency of a correlation between higher opioid dosages and increased laxative use; however, further research is needed to explore the specifics of this relationship. In addition, studies have shown that OIC is not dose-dependent [27]. Two possible reasons may explain why more laxatives are prescribed when the dosage of weak opioids increases. Firstly, physicians who prescribe high doses of opioids are likely to be familiar with the side effects of the treatment and might prescribe more laxatives because of their detailed observation of the patient's symptoms of constipation. Secondly, considering the precedence of successful constipation treatment, physicians are likely to prescribe high doses of opioids. This study investigated prescription trends, and it is unclear whether or not the patients indeed developed OIC. Hence, future studies are required to investigate the relationship between weak opioid use and the development of OIC.

Magnesium oxide is the preferred osmotic laxative in Japan for OIC management [28]. Naldemedine is approved for OIC; however, per the Japanese guidelines, it is recommended if conventional laxatives (osmotic or stimulant) are inadequate in managing OIC [20]. This is consistent with the American Gastroenterology Association guidelines [17] and the European expert consensus statement [18]. Our findings revealed that magnesium oxide was most prescribed (2,320 (66.6%)), followed by sennoside (484 (13.9%)) and naldemedine (301 (8.6%)) at week 1. Over 12 weeks, while the proportion of magnesium oxide gradually decreased to 55.2%, that of naldemedine and other laxatives increased to 13.8% and 9.0%, respectively. These results demonstrate adherence to the prescription guidelines in Japan [19].

The median and average amount of weak opioids tended to be higher in patients with laxatives than in those without laxatives from the second week. In this study, the number of patients receiving laxatives decreased over time, and among patients without laxatives, the median tramadol dose was <100 mg, which was lower than the recommended initial dose of weak opioids. It is possible that patients who were avoiding constipation by reducing tramadol use could be classified in the group without laxatives.

We used Japan's largest health insurance claims database in the current study, a validated real‑world data source [22]. The use of real-world data to observe actual prescription trends makes the findings more generalizable to the broader population of Japanese patients with non-cancer pain and OIC. In addition, the large sample size contributes to the robustness of the findings. While most studies on OIC focus on cancer-related pain or the use of strong opioids, this study focused on the use of weak opioids for non-cancer pain. Given that this population may exhibit different laxative prescription patterns for OIC when compared with those observed for cancer-related pain, the study fills an important research gap.

The study has several limitations. Since this is a health insurance claims database study using disease conditions listed on medical receipts, disease misclassification is possible, as the actual patient condition may differ from the classification based on the insurance-related terms. It is unclear whether or not the patients developed OIC. In addition, since the study relies entirely on the prescription data, the lack of clinical symptom data could introduce bias, as it was assumed that all laxative prescriptions were due to OIC and not due to another indication. Alternatively, it is also possible that some patients with OIC might not have been prescribed laxatives. The study was limited to patients prescribed weak opioids continuously to ensure their intake. Therefore, patients who discontinued weak opioids due to their side effects, including OIC, were excluded. Additionally, the study only included patients who were not on any laxatives in the past three months, creating a relatively constipation-resistant group of patients that may not fully represent the OIC burden in patients with chronic non‑cancer pain. Moreover, data on adults aged ≥65 years are limited in the JMDC database. As both constipation and opioid use are prevalent among older adults, this may have resulted in an underestimation of OIC prevalence in the broader population. Overall, these factors may limit the generalizability of this study. Furthermore, the patients' symptoms of constipation are unknown. As the prescription details are based on the claims database, possible bias due to incorrect data entry cannot be denied. Further, it is not possible to ascertain whether the patient followed the drug regimen (weak opioids and laxatives) and was adherent to treatment per prescription. We also did not consider the use of over-the-counter laxatives. Since some patients would prefer over-the-counter options for various reasons (e.g., convenience, cost, accessibility), the actual use of laxatives may be underrepresented in our study. Since we excluded patients receiving laxatives three months before study initiation, the data provide information on prophylactic and therapeutic laxative use in OIC. We evaluated laxative prescriptions over 12 weeks, which could help us understand the laxative prescription trends and rates. This study provides insights into early prescription patterns of laxatives for OIC in younger Japanese patients (<65 years of age) with chronic non-cancer pain. Further research is warranted to confirm these patterns in older populations (≥65 years of age), who are more likely to experience OIC, as well as to understand the long-term management of OIC beyond the initial 12 weeks of therapy.

## Conclusions

This study used the JMDC database to examine the prescription pattern of laxatives for OIC in Japanese patients administered weak opioids for non-cancer pain. While unmeasured factors, such as OTC laxative use and adherence to prescribed medications, may also play a substantial role in OIC management, this study demonstrated for the first time that the actual prescription status tended to be different, depending on the duration after weak opioid initiation and the amount of weak opioids prescribed. Additionally, the fact that opioid usage was less than the standard dosage in the group not using laxatives suggests that if OIC treatment is not well managed, it could potentially interfere with pain management.

## Appendices

Appendix A

**Table 3:** List of pain classifications (ICD-10 classification) ICD-10, International Classification of Diseases, Tenth Revision (https://iris.who.int/handle/10665/42980)

Pain classification	Definition level	ICD-10 classification code	ICD-10 classification name
Lower back pain	ICD-10 middle classification	M40-M43	Degenerative spine disorders
		M45-M49	Spinal disorders
		M50-M54	Other spinal disorders
Joint pain	ICD-10 middle classification	M05-M14	Inflammatory polyarticular disorder
		M15-M19	Arthropathy
		M20-M25	Other joint disorders
		M60-M63	Muscle disorder
		M70-M79	Other soft tissue disorders
Neuropathic pain	ICD-10 subclassification	G98	Neuropathic pain
		G64	Peripheral neuropathic pain
		G969	Central neuropathic pain
		E144	Diabetic neuropathic pain
		B02	Shingles (herpes zoster)
	ICD-10 middle classification	G50-G59	Disorders of nerves, nerve roots, and nerve plexuses
		G60-G64	Polyneuropathy and other peripheral nervous system disorders

Appendix B

**Table 4:** Age distribution of patients n, number of patients

Age (years)	n (%)
Total	42,514 (100)
≤9	18 (0.0)
10 to 19	400 (0.9)
20 to 29	1,855 (4.4)
30 to 39	4,681 (11.0)
40 to 49	11,572 (27.2)
50 to 59	15,469 (36.4)
≥60	8,519 (20.0)

Appendix C

**Table 5:** Breakdown of laxative prescriptions n, number of patients Others: laxatives listed in ATC A06A except the laxatives listed in Table 5 ATC: Anatomical Therapeutic Chemical: https://www.who.int/tools/atc-ddd-toolkit/atc-classification

Week​	1​	2​	3​	4​	5​	6​	7​	8​	9​	10​	11​	12​
Weak opioid use​, n	42,514​	42,514​	42,514​	42,514​	37,482​	29,886​	25,473​	22,064​	19,191​	16,774​	15,009​	13,610​
Laxative use​, n	3,289​	1,797​	1,848​	1,384​	1,181​	845​	722​	598​	530​	439​	398​	372​
Laxative use (cumulative), n​	3,486​	1,991​	2,023​	1,527​	1,312​	936​	820​	681​	587​	487​	450​	420​
Laxative prescriptions, n (%)												
Magnesium oxide	2,320 (66.6)​	1,213 (60.9)​	1,306 (64.6)​	962 (63.0)​	810 (61.7)​	565 (60.4)​	479 (58.4)​	397 (58.3)​	333 (56.7)​	291 (59.8)​	270 (60.0)​	232 (55.2)​
Sennoside	484 (13.9)​	280 (14.1)​	236 (11.7)​	182 (11.9)​	150 (11.4)​	123 (13.1)​	107 (13.0)​	87 (12.8)​	70 (11.9)​	60 (12.3)​	47 (10.4)​	55 (13.1)​
Naldemedine	301 (8.6)​	225 (11.3)​	230 (11.4)​	188 (12.3)​	169 (12.9)​	128 (13.7)​	104 (12.7)​	93 (13.7)​	93 (15.8)​	65 (13.3)​	62 (13.8)​	58 (13.8)​
Others	207 (5.9)​	135 (6.8)​	112 (5.5)​	92 (6.0)	92 (7.0)​	54 (5.8)​	63 (7.7)​	49 (7.2)​	46 (7.8)​	36 (7.4)​	43 (9.6)​	38 (9.0)​
Senna	65 (1.9)​	42 (2.1)​	61 (3.0)​	35 (2.3)​	38 (2.9)​	24 (2.6)​	25 (3.0)​	20 (2.9)​	22 (3.7)​	14 (2.9)​	8 (1.8)​	14 (3.3)​
Glycerin enema​	47 (1.3)	38 (1.9)​	25 (1.2)​	18 (1.2)	11 (0.8)​	9 (1.0)​	10 (1.2​)	5 (0.7)​	2 (0.3)​	3 (0.6)​	5 (1.1)​	7 (1.7)​
Kampo	25 (0.7)​	25 (1.3)​	30 (1.5)​	25 (1.6)​	21 (1.6)​	14 (1.5)​	17 (2.1)​	15 (2.2)​	10 (1.7)​	7 (1.4)​	7 (1.6​)	9 (2.1)​
Elobixibat	19 (0.5)​	12 (0.6)​	9 (0.4)​	14 (0.9)​	8 (0.6)​	8 (0.9​)	6 (0.7)​	7 (1.0)​	4 (0.7)​	7 (1.4)​	5 (1.1)​	5 (1.2)​
Bisacodyl	14 (0.4)​	12 (0.6​)	6 (0.3)​	2 (0.1)​	3 (0.2)​	5 (0.5)​	2 (0.2)​	3 (0.4)​	3 (0.5)​	1 (0.2)​	0 (0.0)​	0 (0.0)​
Lactulose	2 (0.1)​	2 (0.1)​​	2 (0.1)​​	1 (0.1)​	2 (0.2)​	0 (0.0)​	2 (0.2)​	0 (0.0)​	1 (0.2)​	0 (0.0)​	2 (0.4)​	0 (0.0)​
Linaclotide	2 (0.1)​​	7 (0.4)​	6 (0.3)​	8 (0.5)​	8 (0.6)​	6 (0.6)​	5 (0.6)​	4 (0.6)​	3 (0.5)​	3 (0.6)​	1 (0.2)​	2 (0.5)​
Sodium picosulphate	0 (0.0)​​	0 (0.0)​​	0 (0.0)​​	0 (0.0)​​	0 (0.0)​​	0 (0.0)​​	0 (0.0)​​	0 (0.0)​​	0 (0.0)​​	0 (0.0)​​	0 (0.0)​​	0 (0.0)​​
Lubiprostone	0 (0.0)​​	0 (0.0)​​	0 (0.0)​​	0 (0.0)​​	0 (0.0)​​	0 (0.0)​​	0 (0.0)​​	0 (0.0)​​	0 (0.0)​​	0 (0.0)​​	0 (0.0)​​	0 (0.0)​​
Polyethylene glycol	0 (0.0)​​	0 (0.0)​​	0 (0.0)​​	0 (0.0)​​	0 (0.0)​​	0 (0.0)​​	0 (0.0)​​	0 (0.0)​​	0 (0.0)​​	0 (0.0)​​	0 (0.0)​​	0 (0.0)​​
Other osmotic laxative​s	0 (0.0)​​	0 (0.0)​​	0 (0.0)​​	0 (0.0)​​	0 (0.0)​​	0 (0.0)​​	0 (0.0)​​	0 (0.0)​​	0 (0.0)​​	0 (0.0)​​	0 (0.0)​​	0 (0.0)​​
Other stimulant laxatives	0 (0.0)​​	0 (0.0)​​	0 (0.0)​​	0 (0.0)​​	0 (0.0)​​	0 (0.0)​​	0 (0.0)​​	1 (0.1)​	0 (0.0)​​	0 (0.0)​​	0 (0.0)​​	0 (0.0)​​
